# The link between initial cardiac rhythm and survival outcomes in in-hospital cardiac arrest using propensity score matching, adjustment, and weighting

**DOI:** 10.1038/s41598-024-58468-y

**Published:** 2024-04-01

**Authors:** Yong Han, Haofei Hu, Yuankai Shao, Zhe Deng, Dehong Liu

**Affiliations:** 1 Department of Emergency, Shenzhen Second People’s Hospital, The First Affiliated Hospital of Shenzhen University, No.3002 Sungang Road, Futian District, Shenzhen, 518035 Guangdong Province China; 2 Department of Nephrology, Shenzhen Second People’s Hospital, Shenzhen, 518035 Guangdong Province China

**Keywords:** Shockable rhythm, Inverse probability of treatment weighted, Propensity score matching, In-hospital cardiac arrest, Standardized mortality ratio weighted, Cardiology, Diseases, Signs and symptoms

## Abstract

The association between the initial cardiac rhythm and short-term survival in patients with in-hospital cardiac arrest (IHCA) has not been extensively studied despite the fact that it is thought to be a prognostic factor in patients with out-of-hospital cardiac arrest. This study aimed to look at the relationship between initial shockable rhythm and survival to hospital discharge in individuals with IHCA. 1516 adults with IHCA who received chest compressions lasting at least two minutes at the National Taiwan University Hospital between 2006 and 2014 made up the study population. Propensity scores were estimated using a fitted multivariate logistic regression model. Various statistical methodologies were employed to investigate the association between shockable rhythm and the probability of survival to discharge in patients experiencing IHCA, including multivariate adjustment, propensity score adjustment, propensity score matching, and logistic regression based on propensity score weighting. In the original cohort, the multivariate-adjusted odds ratio (OR) was 2.312 (95% confidence interval [CI]: 1.515–3.531, *P* < 0.001). In additional propensity score adjustment, the OR between shockable rhythm and the probability of survival to hospital discharge in IHCA patients was 2.282 (95% CI: 1.486, 3.504, *P* < 0.001). The multivariate-adjusted logistic regression model analysis revealed that patients with shockable rhythm had a 1.761-fold higher likelihood of surviving to hospital release in the propensity score-matched cohort (OR = 2.761, 95% CI: 1.084–7.028, *P* = 0.033). The multivariate-adjusted OR of the inverse probability for the treatment-weighted cohort was 1.901 (95% CI: 1.507–2.397, *P* < 0.001), and the standardized mortality ratio-weighted cohort was 2.692 (95% CI: 1.511–4.795, *P* < 0.001). In patients with in-hospital cardiac arrest, Initial cardiac rhythm is an independent predictor of survival to hospital discharge. Depending on various statistical methods, patients with IHCA who have a shockable rhythm have a one to two fold higher probability of survival to discharge than those who have a non-shockable rhythm. This provides a reference for optimizing resuscitation decisions for IHCA patients and facilitating clinical communication.

## Introduction

Despite significant improvements in preventive measures, sudden cardiac arrest continues to be a serious issue for public health^1^. Cardiovascular arrest is a medical emergency that almost always results in death; each year, approximately 290,000 in-hospital cardiac arrests and 350,000 out-of-hospital cardiac arrests occur in the United States^2^^,^^3^. Traditionally, in-hospital cardiac arrest (IHCA) has been viewed as a condition with a very poor prognosis, and resuscitation may not even be required^2^. However, recent data suggest that this situation has improved. This improvement might be attributed to a better comprehension of the effects clinical treatment can have on patients who have had IHCA and cardiac arrest in general^4^^,^^5^. Despite the increased interest, compared to out-of-hospital cardiac arrest(OHCA) and other cardiovascular conditions, such as stroke and myocardial infarction(MI), IHCA still receives less attention^2^. The necessity of concentrating on both IHCA and OHCA in attempts to increase cardiac arrest patient survival must be emphasized.

Cardiac arrest may be due to pulseless ventricular tachycardia (PVT), ventricular fibrillation (VF), asystole (ASY), or pulseless electrical activity (PEA)^6^^,^^7^. ASY and PEA are non-shockable rhythms, while VF and PVT are shockable. The two factors that have been shown to be most strongly associated with the prognosis of patients in cardiac arrest are cardiac rhythm and the duration of the cardiac arrest^8–10^. Some studies have found that patients in shockable rhythm have a better prognosis compared to patients in cardiac arrest with non-shockable rhythm^11–14^. However, these studies have focused on OHCA. Few studies have examined the relationship between shockable rhythm and the prognosis of patients with IHCA ^15^. In addition, conventional parsimonious regression models based on analytical adjustments were mostly utilized to control for confounders in previous studies. However, such models could still produce bias as a result of residual or unmeasured model confounding and overfitting^16^^,^^17^. Propensity score (PS)-based research methods are regarded as the primary alternative for reducing confounding in observational studies. Both small-sample and large-sample theories show that adjustment for the scalar PS is adequate to remove bias brought on by observed covariables^18^^,^^19^. Several propensity-score methods have been proposed to control for confounding, including propensity adjustment, propensity matching, and propensity-based weighting^19–21^.

Therefore, based on the current state of research on the impact of cardiac rhythm on the prognosis of patients with IHCA, no study has applied PS-based methods to examine this relationship. Utilizing data published from an observational cohort study, we conducted a second analysis to investigate the impact of cardiac rhythm on the short-term prognosis of IHCA by various statistical models.

## Methods

### Study design

This is a secondary analysis of a retrospective study cohort established by Chih-Hung Wang et al.^22^. The target independent variable was cardiac rhythm in patients with IHCA. The outcome variable was survival to hospital discharge in patients with IHCA.

### Data source

The original data were obtained free of charge from an open-access journal, PLOS ONE, and provided by Wang et al. ^22^. The dataset was obtained from a published article-Outcomes of Adult In-Hospital Cardiac Arrest Treated with Targeted Temperature Management: A Retrospective Cohort Study (https://doi.org/10.1371/journal.pone.0166148) ^22^. According to the Creative Commons Attribution Non-Commercial (CC BY-NC 4.0) license, this open-access article may be shared, remixed, modified, and used to generate derivative works as long as the author and source are attributed^22^. We appreciate the authors providing the data, and we express our gratitude here.

### Study population

The original researchers screened patients who suffered IHCA and had chest compressions of greater than or equal to 2 min in duration at the National Taiwan University Hospital between 2006 and 2014 ^22^. The Institutional Review Board of National Taiwan University Hospital gave its clearance for the original study’s conduct and waived the need for patient consent (Reference number: 201601045RINB)^22^. In addition, the study was approved by the Institutional Review Board of Shenzhen Second People's Hospital. Furthermore, the original research was done in accordance with the Declaration of Helsinki. All techniques were carried out in compliance with the relevant guidelines and regulations, as stated in the declarations Sect. ^22^. So did this secondary analysis.

**Figure 1:**
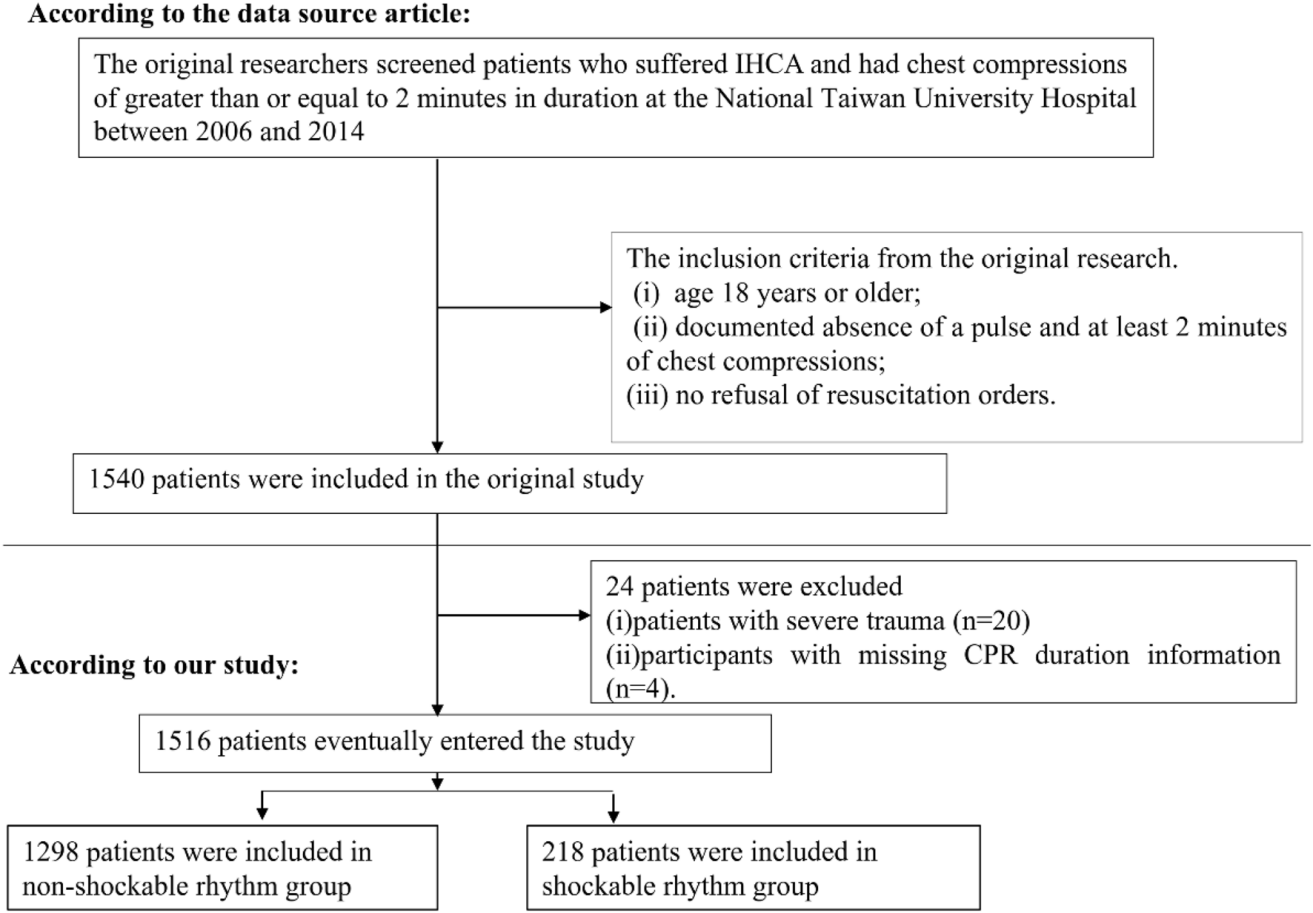
Flowchart of study participants.

The initial study included patients who suffered from IHCA at the National Taiwan University Hospital between 2006 and 2014. The following were the inclusion criteria from the original research. (i) age 18 years or older; (ii) no refusal of resuscitation orders; (iii) documented absence of a pulse and at least 2 min of chest compressions. Finally, The original study ultimately comprised 1,540 patients. In the present investigation, patients with severe trauma (n = 20) and lacking CPR duration data (n = 4) were eliminated. Finally, 1,516 individuals were included in our study for secondary analysis. The procedure for selecting the participants is shown in Fig. 1.

### Initial cardiac rhythm

Cardiac rhythm was recorded as a dichotomous variable (shockable rhythm and non-shockable rhythm). The initial cardiac rhythm was defined as the rhythm initially monitored according to the Utstein template after IHCA ^7^^,^^22^. ASY or PEA was classified as a non-shockable rhythm, while PVT or VF was characterized as a shockable rhythm.

### Outcome measures

The primary outcome was survival status at hospital discharge. It was a dichotomous variable (0 = mortality, 1 = survival).

### Covariates

The original researchers recorded the following information for patients with IHCA: age, sex, comorbidities, key treatments performed during cardiac arrest or after a sustained return of spontaneous circulation (ROSC), and vital signs after sustained ROSC^22^. Our study’s variables were chosen in accordance with prior research and our clinical expertise. Covariates included the following: (i)categorical variables: sex, acute myocardial infarction (AMI), heart failure, arrhythmia history, renal insufficiency, respiratory insufficiency, hypotension, regular dialysis, hepatic insufficiency, diabetes, metabolic or electrolyte abnormality, pneumonia, metastatic cancer or any blood-borne malignancy, bacteremia, thrombocytopenia, intracranial hemorrhage, acute stroke, arrest location, arrest at night, arrest on the weekend, witnessed arrest, mechanical ventilation, antiarrhythmics, vasopressors, intra-aortic balloon pumping, and percutaneous coronary intervention, extracorporeal cardiopulmonary resuscitation (ECPR). (ii) continuous variables: age (years) , CPR duration(minutes). In the current study, there were only 2 participants with missing regular dialysis information. We included them as a separate category of regular dialysis. That is, regular dialysis was classified as: dialysis, not regular dialysis, and not recorded.

### Statistical analysis

Participants were categorized according to cardiac rhythm, and continuous data were reported as means standard deviations (normal distribution) or medians (quartiles) (skewed distribution), and categorical variables were expressed as frequencies or percentages. We utilized the Kruskal–Wallis H-test (skewed distribution), the independent samples t-test (normal distribution), or the chi-square test (categorical variables) to test for differences between the various groups of cardiac rhythms.

Our goal was to investigate the association between initial cardiac rhythm and short-term survival in patients with IHCA using various confounding factor-controlling techniques. We specifically used four propensity score approaches and multivariable-adjusted logistic regression models, totaling five methods. Inverse probability of treatment weighting (IPTW), PS-adjusted logistic regression models, PS matching multivariable-adjusted logistic regression models, and standardized mortality rate (SMR)-weighted multivariable-adjusted logistic regression models were the four propensity score methods.

With cardiac rhythm as the independent variable and all baseline parameters given in Table 1 as variables, PS was calculated using a non-parsimonious multivariable logistic regression model. The variables used for matching included sex, heart failure, AMI, arrhythmia history, renal insufficiency, respiratory insufficiency, hypotension, regular dialysis, hepatic insufficiency, bacteremia, pneumonia, metabolic or electrolyte abnormality, diabetes, metastatic cancer or any blood-borne malignancy, thrombocytopenia, intracranial hemorrhage, acute stroke, arrest on the weekend, arrest at night, arrest location, witnessed arrest, mechanical ventilation, antiarrhythmics, vasopressors, intra-aortic balloon pumping, percutaneous coronary intervention, age, and CPR duration; matching using 1:1 matching scheme with no replacement (greedy matching algorithm) and caliper width equal to 0.001^23–26^. Multivariate logistic regression was applied to analyze the association between variables with differences between shockable and non-shockable rhythm groups in the PS-matched cohort and survival to hospital discharge among patients with IHCA.

**Table 1:** Baseline characteristics before and after propensity score matching.

Monitored rhythm	Before matching			After matching		*P*-value
	Non-shockable	Shockable	*P*-value	Non-shockable	Shockable	
N	1298	218		161	161	
Age(years)	65.074 ± 16.823	66.177 ± 16.238	0.368	65.645 ± 16.736	65.243 ± 17.115	0.832
CPR duration(minutes)	35.547 ± 35.859	28.789 ± 38.614	< 0.001	61.845 ± 42.571	30.689 ± 42.482	< 0.001
ECPR	91 (7.011%)	24 (11.009%)	0.039	15 (9.317%)	16 (9.938%)	0.850
Male	780 (60.092%)	139 (63.761%)	0.305	104 (64.596%)	101 (62.733%)	0.728
Heart failure	300 (23.112%)	85 (38.991%)	< 0.001	66 (40.994%)	54 (33.540%)	0.167
AMI	157 (12.096%)	62 (28.440%)	< 0.001	37 (22.981%)	34 (21.118%)	0.687
Arrhythmia	203 (15.639%)	61 (27.982%)	< 0.001	44 (27.329%)	40 (24.845%)	0.612
Hypotension	301 (23.190%)	56 (25.688%)	0.421	30 (18.634%)	42 (26.087%)	0.108
Respiratory insufficiency	945 (72.804%)	148 (67.890%)	0.134	106 (65.839%)	114 (70.807%)	0.338
Renal insufficiency	531 (40.909%)	96 (44.037%)	0.386	65 (40.373%)	75 (46.584%)	0.261
Regular dialysis			0.063			0.785
**Yes**	210 (16.179%)	49 (22.477%)		33 (20.497%)	35 (21.739%)	
**No**	1086 (83.67%)	169 (77.52%)		128(79.503)	126(78.261)	
**Not recorded**	2 (0.15%)	0				
Hepatic insufficiency	247 (19.029%)	29 (13.303%)	0.043	21 (13.043%)	25 (15.528%)	0.524
Metabolic or electrolyte abnormality	219 (16.872%)	38 (17.431%)	0.839	19 (11.801%)	33 (20.497%)	0.034
Diabetes	402 (30.971%)	92 (42.202%)	0.001	64 (39.752%)	57 (35.404%)	0.421
Pneumonia	428 (32.974%)	60 (27.523%)	0.111	48 (29.814%)	48 (29.814%)	1.000
Bacteremia	106 (8.166%)	19 (8.716%)	0.785	14 (8.696%)	16 (9.938%)	0.701
Metastatic cancer or any blood borne malignancy	316 (24.345%)	22 (10.092%)	< 0.001	18 (11.180%)	22 (13.665%)	0.499
Thrombocytopenia	157 (12.096%)	23 (10.550%)	0.514	7 (4.348%)	19 (11.801%)	0.014
Intracranial haemorrhage	32 (2.465%)	2 (0.917%)	0.153	1 (0.621%)	2 (1.242%)	0.562
Acute stroke	56 (4.314%)	11 (5.046%)	0.627	10 (6.211%)	9 (5.590%)	0.813
Arrest at night	816 (62.866%)	127 (58.257%)	0.194	106 (65.839%)	103 (63.975%)	0.726
Arrest on weekend	363 (27.966%)	69 (31.651%)	0.265	55 (34.161%)	53 (32.919%)	0.813
Arrest location			< 0.001			0.333
ICU	557 (42.912%)	129 (59.174%)		78 (48.447%)	89 (55.280%)	
GW	648 (49.923%)	74 (33.945%)		77 (47.826%)	62 (38.509%)	
ED	41 (3.159%)	5 (2.294%)		2 (1.242%)	4 (2.484%)	
Other locations	52 (4.006%)	10 (4.587%)		4 (2.484%)	6 (3.727%)	
Witnessed arrest	877 (67.565%)	172 (78.899%)	< 0.001	115 (71.429%)	121 (75.155%)	0.450
Mechanical ventilation	270 (20.801%)	54 (24.771%)	0.186	42 (26.087%)	38 (23.602%)	0.606
Antiarrhythmics	118 (9.091%)	42 (19.266%)	< 0.001	27 (16.770%)	19 (11.801%)	0.203
Vasopressors	534 (41.140%)	105 (48.165%)	0.052	71 (44.099%)	72 (44.720%)	0.911
Intra-aortic balloon pumping	5 (0.385%)	10 (4.587%)	< 0.001	0 (0.000%)	1 (0.621%)	
Percutaneous coronary intervention	28 (2.157%)	30 (13.761%)	< 0.001	9 (5.590%)	8 (4.969	0.803

Continuous data are summarized as the mean (standard deviation) or median (interquartile range), whilst categorical variables are reported as percentages (%).

*ECPR* Extracorporeal cardiopulmonary resuscitation; *CPR* Cardiopulmonary resuscitation; *ICU* Intensive care unit; *GW* General ward; *ED* Emergency department.

We applied four PS-based statistics as follows. (1) Propensity score adjustment: multivariate-adjusted logistic regression models based on the original cohort (all patients were included in the analysis) with additional adjustment for PS.^24^^,^^27^. (2) We used multivariable-adjusted logistic regression models to assess the relationship between initial cardiac rhythm and survival in patients with IHCA in the PS-matched cohort. (3) The IPTW estimator estimated the treatment effect for a population with the same risk factor distribution as found in all study participants. IPTW was calculated as the inverse of PS as the weight for patients with shockable rhythm(1/PS) and the inverse of 1 minus the PS as the weight for individuals with non-shockable rhythm(1/(1-PS)). The IPTW model was applied to generate the weighted cohort^19^^,^^24^^,^^28^. Inverse probability weighted in accordance with PS and adjusted for the same factors made up the IPTW multivariate-adjusted logistic regression model. All patients were included in the analysis. (4) The SMR-weighted estimator estimates the effect of treatment in a population with a distribution of risk factors equal to that found only in treated study subjects. SMR-weighted analysis used the value 1 as the weight for the shockable rhythm group and divided PS by (1—PS) as the weight for the non-shockable rhythm group (PS/(1—PS)), and estimated standardized effect indicators for the shockable rhythm group (exposed group) as a standard population^19^^,^^29^. SMR-weighted multivariable-adjusted logistic regression models adjusted for the same strata and covariates. All patients were included in the analysis. We adjusted for confounding factors based on clinical experience and literature reports. In addition, it should be emphasized that the multivariate-adjusted logistic regression model was adjusted for the same covariates as the four PS methods. Furthermore, we determined the relationship between PS and initial cardiac rhythm in patients with IHCA using logistic regression with cubic spline function and smoothed curve fitting.

In order to assess how reliable the findings were, we ran a number of sensitivity studies. First, since there was a significant difference between the stratum with low and high intensities in terms of survival OR. Patients with low PS had a lower survival rate. As a result, we eliminated individuals with a PS of less than 0.07 and ran sensitivity analyses using five different models. In addition, we explored the possibility of unmeasured confounders between initial cardiac rhythm and survival to hospital discharge in subjects with IHCA by calculating the E-values ^30^.

STROBE guidelines were followed in the writing of all findings^31^. To conduct the necessary statistical tests, we utilized both Empower Stats (X&Y Solutions, Inc., Boston, MA, http://www.empowerstats.com) and R (http://www.r-project.org, The R Foundation). The cutoff for statistical significance was a P value of 0.05. (two-sided).

### Ethical approval

The original research was done with clearance from the Institutional Review Board of National Taiwan University Hospital, which waived the need for patient consent (Reference number: 201601045RINB).

### Patient and public involvement

Patients and/or the public were not involved in the design, or conduct, or reporting, or dissemination plans of this research.

## Results

### Characteristics of participants

A total of 1,516 individuals were included in the analysis (60.62% men and 39.38% women). Of these, 218 (14.38%) were shockable rhythms, and 1,298 (85.62%) were non-shockable. The mean age was 65.23 ± 16.74 years old. PS was estimated using a fitted multivariate logistic regression model, with cardiac rhythm serving as the independent variable. Before PS matching, most baseline characteristics differed between the shockable rhythm and non-shockable rhythm groups (*p* < 0.05) (Table 1). Compared to patients with non-shock rhythm, patients with shock rhythm had higher rates of heart failure, AMI, arrhythmia, regular dialysis, diabetes, witnessed arrest, antiarrhythmics, intra-aortic balloon pumping, and percutaneous coronary intervention, and lower rates of hepatic insufficiency. In addition, the duration of CPR was shorter in the shock rhythm group. 161 patients with IHCA in a shockable rhythm and 161 patients with IHCA in a non-shockable rhythm were successfully matched after one-to-one matching using PS analysis. After matching, the discrepancies between the non-shockable-rhythm and shockable-rhythm groups were not statistically significant for almost all baseline variables, with the exception of CPR duration, suggesting that the PSs were properly matched. That is, there were only minor variations in baseline features between the shockable and non-shockable rhythm groups after matching (Table 1). In addition, the link between PS and cardiac rhythm in participants with IHCA was determined using logistic regression with cubic spline function and smoothed curve fitting. It was found that a higher PS was linked to a higher probability of shockable rhythm in individuals with IHCA (Supplementary Figure S1).

**Figure 2:**
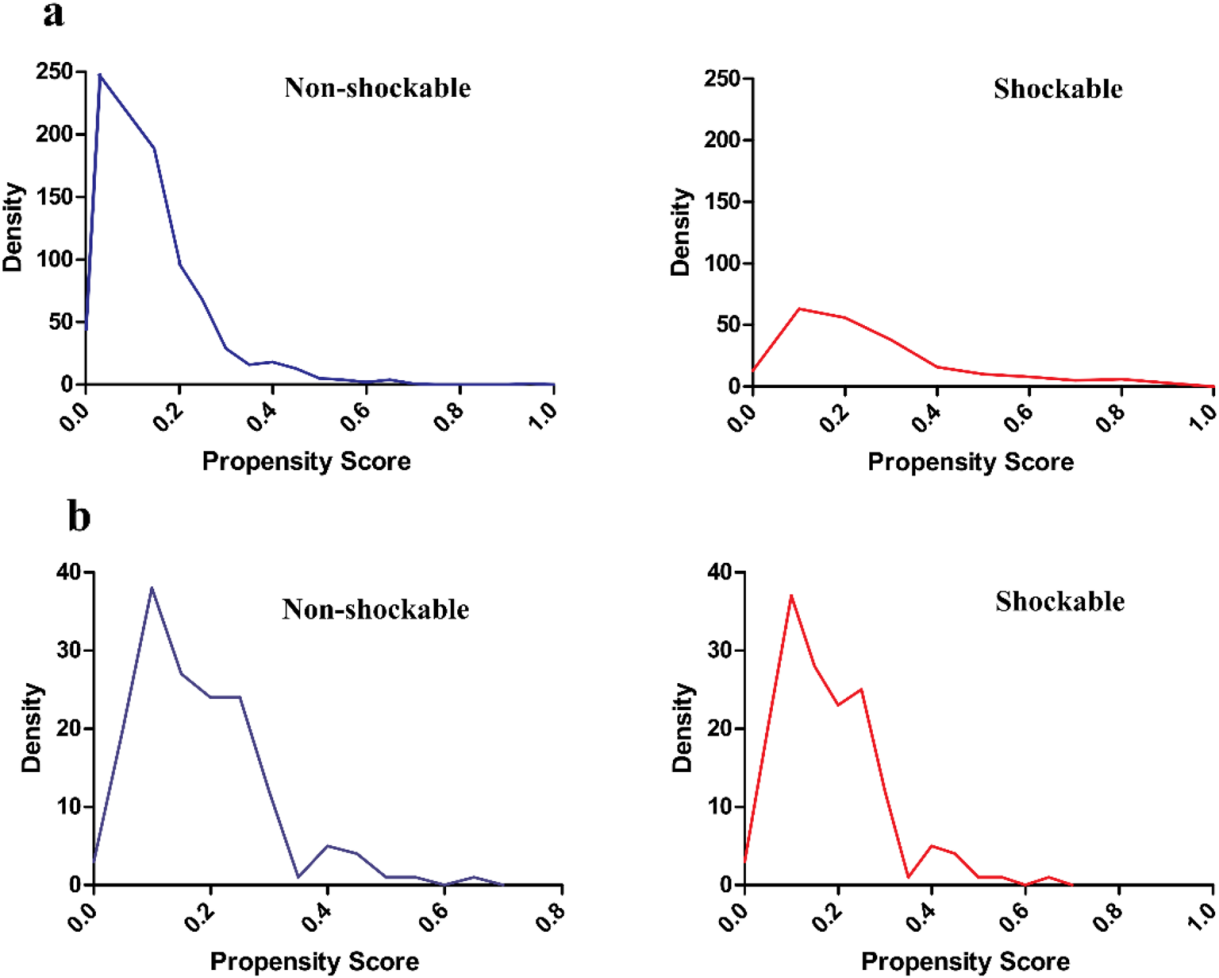
Distribution of propensity score in the shockable and non-shockable rhythm groups before and after matching.

Figure 2a summarized the probability density functions of the PS for individuals with shockable rhythm and non-shockable before matching. As anticipated, the distribution of PS for individuals with non-shockable rhythm changed slightly toward 0, and those with shockable rhythm shifted somewhat toward 1. The figure also demonstrated that the PS for shockable and non-shockable rhythm groups only overlap to a narrow degree. In addition, Fig. 2b summarized the probability density functions of the PS for shockable and non-shockable rhythm participants after matching. The PS distribution for shockable and non-shockable rhythm patients remained basically the same.

Figure 2a showed the probability density functions of the propensity score for participants with shockable and non-shockable rhythms before matching.

Figure 2b showed the probability density functions of the propensity score for participants with shockable and non-shockable rhythms after matching.

### Propensity-stratum-specific effects

In terms of survival rate before hospital discharge, the PS gradients between shockable rhythm and non-shockable rhythm groups were substantial and shockingly distinct. Based on the percentiles of the PS, Supplementary Table S1 displayed the proportions of survival rate prior to hospital release for shockable rhythm and non-shockable rhythm groups. Several points are noteworthy. First, very few members of the group with shockable rhythms had an overall PS below the 30th percentile. Secondly, the survival rate of both groups increased as the PS climbed. The empirical odds ratio for survival rate prior to hospital discharge rose from 1.083 at the 40-50th percentile of the PS to 12.083 at the 99th percentile (Supplementary Table S1).

### Survival rate before hospital discharge

Table 2 displayed the survival rate of patients before hospital discharge in shockable and non-shockable rhythm groups both before and after PS matching. A total of 219 participants survived to hospital discharge before PS matching. The corresponding survival rates in the shockable and non-shockable rhythm groups were 11.4% (95% confidence interval (CI): 9.07–13.13) and 32.57%(95%CI:26.30–38.34), respectively. After the PS-matching, the survival rates of the two groups altered; the survival rates in the shockable and non-shockable rhythm groups were 8.08%(95% CI: 3.82–12.33) and 26.09% (95% CI:19.23–32.94).

**Table 2:** Survival rate before hospital discharge before and after propensity-score matching.

Variable	Participants(n)	Number of survivors (n)	Survival rate (95% CI)(%)
	Before matching		
Total	1516	219	14.45 (12.74–16.22)
Non-shockable	1298	148	11.40(9.07–13.13)
Shockable	218	71	32.57(26.30–38.34)
	After matching		
Total	322	55	17.08(12.95–21.21)
Non-shockable	161	13	8.08(3.82–12.33)
Shockable	161	42	26.09(19.23–32.94)

*CI* Confidence interval.

### The associations of CPR duration, thrombocytopenia, and metabolic or electrolyte abnormalities with survival to hospital discharge

Following PS matching, differences were identified in CPR duration, thrombocytopenia, and metabolic or electrolyte abnormalities between the groups with shockable and non-shockable rhythms. Consequently, a multivariate logistic regression model was applied to both the original and the PS-matched cohorts to further investigate the associations of CPR duration, thrombocytopenia, and metabolic or electrolyte abnormalities with survival to hospital discharge. It was found that no significant associations existed between thrombocytopenia and metabolic or electrolyte abnormalities and survival to discharge in patients experiencing IHCA. However, a negative association was observed between CPR duration and survival to hospital discharge in IHCA patients, with OR (95%CI) of 0.938 (0.927, 0.950) and 0.936 (0.911, 0.961) in the original and PS-matched cohorts, respectively**(**Supplementary Table S2). Therefore, CPR duration was included in all multifactorial regression models that were used to explore the relationship between initial cardiac rhythm and survival to hospital discharge in patients with IHCA.

### Analyze outcomes using various confounding control approaches

We utilized logistic regression models to determine the relationship between cardiac rhythm and the probability of survival to hospital discharge among patients with IHCA in the original, PS-matched, and weighted cohorts. The shockable rhythm was significantly linked with the probability of survival to hospital discharge (OR = 4.672; 95%CI: 3.578–6.100; *P* < 0.001). Specifically, patients with shockable rhythm had a 3.672-fold greater probability of survival to hospital discharge than those with non-shockable rhythm. The link remained after multivariate adjustment (OR = 2.312, 95% CI: 1.515–3.531, *P* < 0.001). In PS adjustment (the adjusted variables were the multivariate-adjusted variable plus PS), the OR between shockable rhythm and the probability of survival to hospital discharge was 2.282 (95% CI: 1.486, 3.504, *P* < 0.001).

Second, the multivariate-adjusted logistic regression model analysis of the PS-matched cohort revealed that the OR between shockable rhythm and the probability of surviving to hospital discharge was 2.761 (95% CI: 1.084–7.028, *P* = 0.033).In addition, in the weighted cohort, the SMR-weighted analysis after multivariate adjustment yielded an OR of 2.692 (95% CI: 1.511–4.795, *P* < 0.001), and the IPTW multivariate-adjusted logistic regression model analysis revealed that the OR between shockable-rhythm and the probability of survival to hospital discharge was 1.901 (95% CI: 1.507–2.397, *P* < 0.001). It must be underlined that the adjusted variables were identical in the PS matching multivariate adjustment, original cohort multivariate adjustment, SMR-weighted and IPTW multivariate adjustment, including age, ECPR, hypotension, sex, HF, MI, hepatic insufficiency, arrhythmia history, renal insufficiency, respiratory insufficiency, regular dialysis, diabetes, thrombocytopenia, metabolic or electrolyte abnormality, pneumonia, bacteremia, cancer, intracranial hemorrhage, acute stroke, arrest location, arrest on the weekend, arrest at night, witnessed arrest, intra-aortic balloon pumping, percutaneous coronary intervention, and CPR duration. Adjusted variables in PS adjustment comprised PS and other model-adjusted variables. (Table 3).

**Table 3:** Relationship between initial monitored rhythm and the probability of short-term survival in patients with IHCA in the crude analysis, multivariable analysis, and four propensity-score methods analyses.

Logistic regression model	Adjusted variables	No	OR	95%CI	*P* value
Crude		1516	4.672	3.578, 6.100	< 0.001
Multivariable-adjusted model	Multivariable†	1516	2.312	1.515, 3.531	< 0.001
Propensity score adjustment	Propensity score + Multivariable†	1516	2.282	1.486, 3.504	< 0.001
Propensity score matching	multivariable†	322	2.761	1.084, 7.028	0.033
IPTW	Multivariable†	1516	1.901	1.507, 2.397	< 0.001
SMR–weighted	multivariate†	1516	2.692	1.511, 4.795	< 0.001

*OR* Odds ratio; *CI* Confidence interval; *IPTW* Inverse-probability-of-treatment weighted; *SMR* Standardized mortality ratio. *IHCA***,** In-hospital cardiac arrest.

Multivariable†: Adjusted for ECPR, age, sex, HF, MI, arrhythmia history, hypotension, respiratory insufficiency, renal insufficiency, regular dialysis, hepatic insufficiency, metabolic or electrolyte abnormality, diabetes, pneumonia, bacteremia, cancer, intracranial hemorrhage, acute stroke, arrest at night, thrombocytopenia, arrest on the weekend, arrest location, witnessed arrest, intra-aortic balloon pumping, percutaneous coronary intervention, and CPR duration.

### Sensitivity analysis

We considered a significant difference in the associated empirical OR for the probability of survival to hospital discharge in patients with IHCA between the participants with low and high PSs (Supplementary Table S1). Therefore, patients whose PS was greater than 0.07 were further examined, the restricted population's crude OR was 3.857 (95% CI: 2.794–5.323, *P* < 0.001). The five methods based on the restricted population yielded ORs similar to those based on the all-population analysis. In the restricted population of the original cohort, the multivariate-adjusted OR was 2.407 (95% CI: 1.549–3.739, *P* < 0.001). In PS adjustment, the results revealed that the OR between shockable rhythm and the probability of survival to hospital discharge was 2.357 (95% CI: 1.509- 3.679, *P* < 0.001). The multivariate-adjusted OR was 2.951 (95% CI: 1.119–7.782, *P* = 0.029) in the PS-matched cohort. The multivariate-adjusted OR of the SMR-weighted and IPTW cohort was 2.888 (95% CI: 1.582–5.272, *P* < 0.001) and 2.154 (95% CI: 1.649, 2.813, *P* < 0.001), respectively (Table 4).

**Table 4:** Relationship between initial monitored rhythm and short-term survival probability in IHCA patients with PS ≥ 0.07 analyzed based on binary logistic regression models and four propensity score methods.

Logistic regression model	Adjusted variables	No	OR	95%CI	*P*
Crude		1063	3.857	2.794, 5.323	< 0.001
Multivariable-adjusted model	Multivariable†	1063	2.407	1.549, 3.739	< 0.001
Propensity score adjustment	Propensity score + Multivariable†	1063	2.357	1.509, 3.679	< 0.001
Propensity score matching	Multivariable†	284	2.951	1.119,7.782	0.029
IPTW	Multivariable†	1063	2.154	1.649, 2.813	< 0.001
SMR-weighted	Multivariable†	1063	2.888	1.582, 5.272	< 0.001

*OR*, Odds ratio; *CI*, Confidence interval; *IPTW*, Inverse-probability-of-treatment weighted; *SMR*, Standardized mortality ratio.

Multivariable†: Adjusted for ECPR, age, sex, HF, MI, arrhythmia history, hypotension, respiratory insufficiency, renal insufficiency, regular dialysis, hepatic insufficiency, metabolic or electrolyte abnormality, diabetes, pneumonia, bacteremia, cancer, intracranial hemorrhage, acute stroke, arrest at night, arrest on the weekend, arrest location, witnessed arrest, intra-aortic balloon pumping, percutaneous coronary intervention, and CPR duration.

In addition, we calculated an E-value to determine the sensitivity to unmeasured confounding variables. The E-value (2.91) was less than the relative risk (9.55) of unmeasured confounders and survival to hospital discharge, indicating that unknown or unmeasured confounders had little influence on the association between shockable rhythm and the probability of survival to hospital discharge. Our principal findings were solid.

## Discussion

This PS-matched cross-sectional study revealed that shockable rhythm was linked to a higher probability of survival before hospital discharge in patients with IHCA. After PS matching, participants with shockable rhythms had a 1.761-fold increase in survival to hospital discharge compared to subjects with non-shockable rhythms. In PS adjustment, the OR between shockable rhythm and survival to hospital discharge was 2.282. In the IPTW and SMR-weighted cohorts, the shockable rhythm was associated with 0.901- and 1.692-fold increases in the likelihood of survival to hospital discharge in patients with IHCA.

We discovered that various confounding factor control strategies produced different ORs. The ORs produced by the original cohort multivariate-adjusted, PS-adjusted, and SMR-weighted multivariate-adjusted logistic regression models were similar. The results of the IPTW multivariate-adjusted logistic regression model were lower. In comparison, the outcomes of the PS matching multivariate-adjusted logistic regression model were marginally higher. Participants with a non-shockable rhythm outnumbered those with a shockable beat by a significant margin. Numerous mismatched individuals with non-shockable rhythms are removed from the analysis. Consequently, the distribution of variables in the (successfully) matched subpopulation would closely resemble that of the treated study group (patients with shockable rhythm). The majority of patients in the shockable rhythm group were in propensity strata with a high probability of survival to hospital discharge, and the SMR-weighted method calculated the average impact of shockable rhythm in a population with a risk factor distribution similar to that of the shockable rhythm group. Consequently, the SMR-weighted estimate’s stronger resemblance to the PS-matched estimate was thus not unexpected.

The IPTW model, in contrast, assessed the average shockable rhythm effect for the total research sample. Given that 90% of the sample population fell under the propensity strata linked with low empirical OR, the IPTW multivariable-adjusted logistic regression model yielded a lower OR. The OR produced by the IPTW multivariate-adjusted logistic regression model was 1.901. By limiting the analysis to the subpopulation of the shockable rhythm and non-shockable rhythm groups with a PS > 0.07, The OR increased to 2.154 in the IPTW multivariate-adjusted logistic regression model. All of these findings indicated that the four PS approaches were able to adjust for confounding variables effectively.

There have been some findings in the past that have shown a significant relationship between shockable rhythm and survival in patients with OCHA. 163 (45%) of OCHA patients with an initial shockable rhythm (n = 360) were alive at discharge in an observational analysis of 1,627 patients. In the subgroup of non-shockable rhythms (n = 1,253), however, 53 (4.2%) individuals remained alive at discharge. Shockable rhythms were associated with a 6.84-fold increase in discharge survival (OR = 7.84, 95% CI: 5.01–12.26) in multivariable-adjusted logistic regression analysis^32^.

In another observational study of sudden cardiac arrest patients in the presence of heart failure, Patients who had shockable rhythm were more likely to survive to be discharged from the hospital (OR: 5.21, 95% CI: 2.99–9.07) than those with non-shockable rhythm after adjusting for sex, age, comorbidities, and race/ethnicity^33^. A recent post hoc analysis of a randomized trial of OHCA in Prague also found a significant association between initial rhythm and survival rate in patients with refractory OHCA. The shockable rhythm was independently related to a lower risk of unfavorable clinical outcome (180-day mortality) (HR = 0.27, 95% CI: 0.18–0.41) ^11^. Our results align with previous studies that demonstrate a positive correlation between shockable rhythm and the likelihood of short-term survival in cardiac arrest patients. However, To the best of our knowledge, the current study is the first to identify a connection between the initial rhythm and survival to hospital discharge in adults with IHCA. In addition, we applied multiple statistical methods to obtain an estimated OR in the range of 1.901–2.761 for the relationship between shockable rhythm and survival to hospital discharge in patients with IHCA, which was much lower than that reported in previous studies.

We analyzed these inconsistent findings for possible explanations: (i) different study populations, including age, sex, and race. Our study population included patients with IHCA, while most other studies were of patients with OHCA. (ii) These studies used a wide variety of samples of different sizes for their analyses. (iii) These studies differed in their adjustment for variables. (iv) Prior research has mostly employed variable adjustment to account for confounders; however, this conventional regression approach may lead to bias owing to residual or unmeasured confounders or overfitting. We used PS combined with regression adjustment to control for confounders and to validate the association between shockable rhythm and the probability of survival to hospital discharge in patients with IHCA. Some investigators have considered shockable rhythm to be a dependent rather than an independent variable and have excluded the initial rhythm from logistic regression models^11^^,^^34^^,^^35^. These studies have shown that the variables contributing to shockable rhythm are similar to the covariates predicting survival (i.e., age, bystander witnessed arrest, bystander CPR). However, the initial shockable rhythm in individuals with IHCA was a standalone predictor of survival in our study. In clinical practice, medical personnel usually have relatively little information about the status of IHCA patients, and initial rhythm is one of the very few parameters that are available and easily identifiable. It provides a reference for optimizing resuscitation decisions for patients with IHCA and facilitates communication with patients' families.

The reasons why patients with initial shockable rhythms typically have a higher probability of survival when receiving CPR are unclear and may be due to the following reasons and mechanisms: First, there is the reversibility of the rhythm. Shockable rhythms, such as VF and PVT, are generally considered to be more “reversible” arrhythmic states. Shock (defibrillation) can directly reverse these arrhythmias and restore normal cardiac rhythm, thereby improving the chances of immediate resuscitation^36^^,^^37^. Besides, during the initial phase of cardiac arrest, the heart and brain are still well oxygenated, and shockable rhythms that can be corrected during this period will naturally result in a higher success rate of cardiac resuscitation. In contrast, non-shockable rhythms usually indicate that the heart is already hypoxemic and energy-depleted, and the chances of recovery are lower^37^^,^^38^. In addition, shockable rhythms indicate that the heart's electrophysiologic activity is still active, meaning that the heart’s muscle cells still have the potential to respond to a shock and resume effective pumping. Non-shockable rhythms, on the other hand, may reflect a severe failure of the electrical activity of the heart’s myocytes, making a recovery, even if attempted with drugs and CPR, highly unsuccessful^39^. Furthermore, shockable rhythms are often associated with specific types of cardiac lesions that may have a better prognosis after successful CPR. For example, VF may be associated with acute myocardial infarction, which may be potentially more favorable to patient survival if it is rapidly identified and treated (including CPR and urgent coronary intervention).

Our study has several advantages. (i) To our knowledge, only a few studies have employed PS matching to investigate the connection between cardiac rhythm and survival until hospital discharge in patients with IHCA. PS-based study methods are considered a core option to control for confounding factors in observational studies. (ii) Since this is a secondary analysis of an observational study, it was susceptible to potential confounding factors. We employed rigorous statistical adjustments to minimize residual confounding interference. Most of the covariates had complete information, and only a few were missing. (iii) The adjustment models we implemented were rarely used. These models include the multivariate-adjusted logistic regression model, PS-adjusted logistic regression model, IPTW, and SMR-weighted multivariate-adjusted logistic regression models. This makes our results relatively more convincing and robust. (iv) Notably, we conducted a series of sensitivity analyses, restricted the study population to patients with PS larger than or equal to 0.07, and generated E-values to investigate the potential of unmeasured confounders. The results showed that the results were reliable.

The following are some of the study’s potential limitations. First of all, this study is a secondary analysis based on publicly available data. This means that variables that are not in the dataset cannot be adjusted. Nevertheless, we assessed the unmeasured confounding effects using the E-value and discovered that our study was steady and dependable. Second, all of the participants were Chinese. Consequently, additional research is needed to confirm whether or whether these findings also hold true for people of different races. Third, after PS matching, discrepancies in CPR duration between shockable and non-shockable rhythm groups persisted. However, we conducted multivariate-adjusted analyses. These analyses imply that our findings are reliable. Fourth, the PS approaches attempt to minimize the impact of the known confounding factors. However, it could not guarantee that all baseline attributes were matched, and the effect of unknown factors was examined. To minimize the impact of some variables in the results, we calibrated the caliper to a width of 0.001. In addition, this observational research gives conclusions about the relationship between shockable rhythm and the probability of survival to hospital discharge but cannot prove a causal relationship between them. Consequently, more prospective investigations are necessary to confirm our results.

## Conclusion

Initial cardiac rhythm is an independent predictor of survival to hospital discharge in patients with IHCA. This study quantified the relationship between shockable rhythm and survival to discharge in patients with IHCA by applying various statistical models and proposed a range of OR values (1.905–2.762). That is, patients with IHCA in the presence of a shockable rhythm have an approximately one- to two fold increased probability of survival to discharge compared with patients with a non-shockable rhythm. This provides a reference for optimizing resuscitation decisions for IHCA patients and facilitating clinical communication.

### Supplementary Information

Supplementary Information.

## Data Availability

The data can be downloaded from the "PLoS One" database. The specific URL is https://doi.org/10.1371/journal.pone.0166148.

## Abbreviations

PVT: Pulseless ventricular tachycardia
VF: Ventricular fibrillation
ASY: Asystole
PEA: Pulseless electrical activity
PS: Propensity score
SMR: Standardised mortality ratio
IPTW: Inverse probability of treatment-weighted
AMI: Acute myocardial infarction
ECPR: Extracorporeal cardiopulmonary resuscitation
OHCA: Out-of-hospital cardiac arrest
CPR: Cardiopulmonary resuscitation
MAR: Missing randomization
IHCA: In-hospital cardiac arrest
CI: Confidence interval
SD: Standardized differences
OR: Odd ratios

## References

[1] Panchal AR (2020). Part 3: adult basic and advanced life support: 2020 american heart association guidelines for cardiopulmonary resuscitation and emergency cardiovascular care. Circulation.

[2] Andersen LW, Holmberg MJ, Berg KM, Donnino MW, Granfeldt A (2019). In-Hospital cardiac arrest: a review. Jama-J. Am. Med. Assoc..

[3] Virani SS (2020). Heart disease and stroke statistics-2020 update: a report from the american heart association. Circulation.

[4] Ofoma UR, Basnet S, Berger A, Kirchner HL, Girotra S (2018). Trends in survival after in-hospital cardiac arrest during nights and weekends. J. Am. Coll. Cardiol..

[5] Chan PS (2016). Resuscitation practices associated with survival after in-hospital cardiac arrest: a nationwide survey. Jama Cardiol..

[6] Kim JG (2021). Prognostic value of changes in the cardiac arrest rhythms from the prehospital stage to the emergency department in out-of-hospital cardiac arrest patients without prehospital returns of spontaneous circulation: a nationwide observational study. PLoS ONE.

[7] Kauppila JP (2018). Association of initial recorded rhythm and underlying cardiac disease in sudden cardiac arrest. Resuscitation.

[8] Aschauer S, Dorffner G, Sterz F, Erdogmus A, Laggner A (2014). A prediction tool for initial out-of-hospital cardiac arrest survivors. Resuscitation.

[9] Nadkarni VM (2006). First documented rhythm and clinical outcome from in-hospital cardiac arrest among children and adults. Jama-J. Am. Med. Assoc..

[10] Rohlin O, Taeri T, Netzereab S, Ullemark E, Djärv T (2018). Duration of Cpr and impact on 30-day survival after rosc for in-hospital cardiac arrest-a swedish cohort study. Resuscitation.

[11] Havranek S, Fingrova Z, Rob D, Smalcova J, Kavalkova P, Franek O, Smid O, Huptych M, Dusik M, Linhart A, Belohlavek J (2022). Initial rhythm and survival in refractory out-of-hospital cardiac arrest. Post-hoc analysis of the prague ohca randomized trial. Resuscitation.

[12] Rajan S (2017). Incidence and survival outcome according to heart rhythm during resuscitation attempt in out-of-hospital cardiac arrest patients with presumed cardiac etiology. Resuscitation.

[13] Mader TJ (2012). Out-of-hospital cardiac arrest outcomes stratified by rhythm analysis. Resuscitation.

[14] Meert KL (2016). Pediatric out-of-hospital cardiac arrest characteristics and their association with survival and neurobehavioral outcome. Pediatr. Crit. Care Med..

[15] Ngunga LM, Yonga G, Wachira B, Ezekowitz JA (2018). Initial rhythm and resuscitation outcomes for patients developing cardiac arrest in hospital: data from low-middle income country. Glob. Heart..

[16] Robins JM, Greenland S (1986). The role of model selection in causal inference from nonexperimental data. Am. J. Epidemiol..

[17] D'Agostino RJ (1998). Propensity score methods for bias reduction in the comparison of a treatment to a non-randomized control group. Stat. Med..

[18] Austin PC (2011). An introduction to propensity score methods for reducing the effects of confounding in observational studies. Multivariate Behav. Res..

[19] Kurth T (2006). Results of multivariable logistic regression, propensity matching, propensity adjustment, and propensity-based weighting under conditions of nonuniform effect. Am. J. Epidemiol..

[20] Han Y (2022). The association between congestive heart failure and one-year mortality after surgery in singaporean adults: a secondary retrospective cohort study using propensity-score matching, propensity adjustment, and propensity-based weighting. Front. Cardiovasc. Med..

[21] Cheng D, Chakrabortty A, Ananthakrishnan AN, Cai T (2020). Estimating average treatment effects with a double-index propensity score. Biometrics.

[22] Wang CH (2016). Outcomes of adult in-hospital cardiac arrest treated with targeted temperature management: a retrospective cohort study. PLoS ONE.

[23] Ahmed A (2006). Heart failure, chronic diuretic use, and increase in mortality and hospitalization: an observational study using propensity score methods. Eur. Heart J..

[24] Geleris J (2020). Observational study of hydroxychloroquine in hospitalized patients with covid-19. N. Engl. J. Med..

[25] Lahmann AL (2020). Predicting factors for long-term survival in patients with out-of-hospital cardiac arrest - a propensity score-matched analysis. PLoS ONE.

[26] Kim K (2021). Impact of controlled normothermia following hypothermic targeted temperature management for post-rewarming fever and outcomes in post-cardiac arrest patients: a propensity score-matched analysis from a multicentre registry. Resuscitation.

[27] McCaffrey DF (2013). A tutorial on propensity score estimation for multiple treatments using generalized boosted models. Stat. Med..

[28] Cole SR, Hernán MA (2008). Constructing inverse probability weights for marginal structural models. Am. J. Epidemiol..

[29] Robins JM, Hernán MA, Brumback B (2000). Marginal structural models and causal inference in epidemiology. Epidemiology.

[30] Haneuse S, VanderWeele TJ, Arterburn D (2019). Using the E-value to assess the potential effect of unmeasured confounding in observational studies. Jama-J. Am. Med. Assoc..

[31] von Elm E (2007). The strengthening the reporting of observational studies in epidemiology (strobe) statement: guidelines for reporting observational studies. Lancet.

[32] Grunau B (2016). Comparing the prognosis of those with initial shockable and non-shockable rhythms with increasing durations of Cpr: informing minimum durations of resuscitation. Resuscitation.

[33] Woolcott OO (2020). Sudden cardiac arrest with shockable rhythm in patients with heart failure. Heart Rhythm.

[34] Stiell IG (1999). Modifiable factors associated with improved cardiac arrest survival in a multicenter basic life support/defibrillation system: opals study phase I results. ontario prehospital advanced life support. Ann. Emerg. Med..

[35] Wilcox-Gök VL (1991). Survival from out-of-hospital cardiac arrest. A Multivariate Anal. Med. Care..

[36] Berdowski J, Berg RA, Tijssen JG, Koster RW (2010). Global incidences of out-of-hospital cardiac arrest and survival rates: systematic review of 67 prospective studies. Resuscitation.

[37] Perkins GD (2015). European Resuscitation council guidelines for resuscitation. Adult basic life support and automated external defibrillatio. Resuscitation.

[38] Soar J (2015). European resuscitation council guidelines for resuscitation. Adult Adv Life Supp Resuscitation.

[39] Kudenchuk PJ (2016). Amiodarone, Lidocaine, or placebo in out-of-hospital cardiac arrest. N. Engl. J. Med..

