# Supplementary material for: The link between initial cardiac rhythm and survival outcomes in in-hospital cardiac arrest using propensity score matching, adjustment, and weighting
Source: Sci Rep. 2024 Apr 1;14:7621. doi: 10.1038/s41598-024-58468-y (PMC10985081; doi:10.1038/s41598-024-58468-y)
Supplement: Supplementary file 1 — Supplementary Information. [file 41598_2024_58468_MOESM1_ESM.docx]

**The Link Between Initial Cardiac Rhythm and Survival Outcomes in In-Hospital Cardiac Arrest Using Propensity Score Matching, Adjustment, and Weighting**

**Running title:** shockable rhythm and Short-term survival rate

Yong Han^1#^, Haofei Hu^2#^, Yuankai Shao^1#^, Zhe Deng^1*^, Dehong Liu^1*^

^1^ Department of Emergency, Shenzhen Second People's Hospital, Shenzhen 518035, Guangdong Province, China

^2^ Department of Nephrology, Shenzhen Second People's Hospital, Shenzhen 518035, Guangdong Province, China

Yong Han^1#^, Haofei Hu^2#^, and Yuankai Shao^1#^ have contributed equally to this work.

*Corresponding author

Zhe Deng

Department of Emergency, Shenzhen Second People's Hospital

No.3002 Sungang Road, Futian District,

Shenzhen 518035,

Guangdong Province,

China.

zdeng_emergency@126.com

*Corresponding author

Dehong Liu

Department of Emergency, Shenzhen Second People's Hospital

No.3002 Sungang Road, Futian District,

Shenzhen 518035,

Guangdong Province,

China

E-mail: dhliu_emergency@163.com

**Table S1 According to the percentile of the propensity score of the entire study population, survival rate before hospital discharge in patients with shockable rhythm and non-shockable rhythm.**

|  | Non-shockable | | | |  | shockable | | | | Empirical OR* |
| --- | --- | --- | --- | --- | --- | --- | --- | --- | --- | --- |
| Percentile |  | No. | Probability of Survival | |  |  |  | Probability of Survival | |  |
|  | Score^*^ |  | No. | % |  | Score^*^ | No. | No. | % |  |
| 0 to 10 | 0.030 | 145 | 6 | 4.14 |  | 0.025 | 7 | 1 | 14.286 |  |
| 10< to <20 | 0.049 | 143 | 11 | 7.69 |  | 0.047 | 8 | 0 |  |  |
| 20< to <30 | 0.062 | 148 | 10 | 6.76 |  | 0.064 | 4 | 0 |  |  |
| 30< to< 40 | 0.078 | 139 | 12 | 8.63 |  | 0.078 | 12 | 2 | 16.67 | 2.117 |
| 40< to <50 | 0.093 | 141 | 24 | 17.02 |  | 0.091 | 11 | 2 | 18.18 | 1.083 |
| 50< to <60 | 0.113 | 133 | 15 | 11.28 |  | 0.112 | 18 | 4 | 22.22 | 2.248 |
| 60< to <70 | 0.139 | 133 | 21 | 15.79 |  | 0.139 | 19 | 2 | 10.53 | 0.627 |
| 70< to <80 | 0.178 | 128 | 16 | 12.50 |  | 0.179 | 23 | 4 | 17.39 | 1.474 |
| 80< to <90 | 0.245 | 107 | 17 | 15.89 |  | 0.243 | 45 | 16 | 35.56 | 2.921 |
| 90< to< 95 | 0.346 | 44 | 8 | 18.18 |  | 0.331 | 32 | 10 | 31.25 | 2.045 |
| 95< to 100 | 0.509 | 37 | 8 | 21.62 |  | 0.611 | 39 | 30 | 76.92 | 12.083 |

Empirical OR: Propensity-stratum-speciﬁc-treatment–mortality odds ratio; Score* : mean propensity score in percentile.

**Table S2** The associations of CPR duration, thrombocytopenia, and metabolic or electrolyte abnormalities with survival to hospital discharge before and after PS matching.

|  | Before matching |  | After matching |
| --- | --- | --- | --- |
|  | (OR.,95%CI) *p* |  | (OR.,95%CI) *p* |
| Thrombocytopenia | 1.270 (0.838, 1.927) 0.260 |  | 0.676 (0.159, 2.884) 0.597 |
| Metabolic or electrolyte abnormality | 0.815 (0.546, 1.218) 0.319 |  | 1.644 (0.576, 4.694) 0.353 |
| CPR duration（minutes） | 0.938 (0.927, 0.950) <0.001 |  | 0.936 (0.911, 0.961) <0.001 |

Note 1: Above model adjusted for ECPR, age, sex, HF, MI, arrhythmia history, hypotension, respiratory insufficiency, renal insufficiency, regular dialysis, hepatic insufficiency, metabolic or electrolyte abnormality, diabetes, pneumonia, bacteremia, cancer, intracranial hemorrhage, acute stroke, thrombocytopenia, arrest at night, arrest on the weekend, arrest location, witnessed arrest, intra-aortic balloon pumping, percutaneous coronary intervention, and CPR duration.

Note 2: When the variable is used as an independent variable, the variable itself was not adjusted.


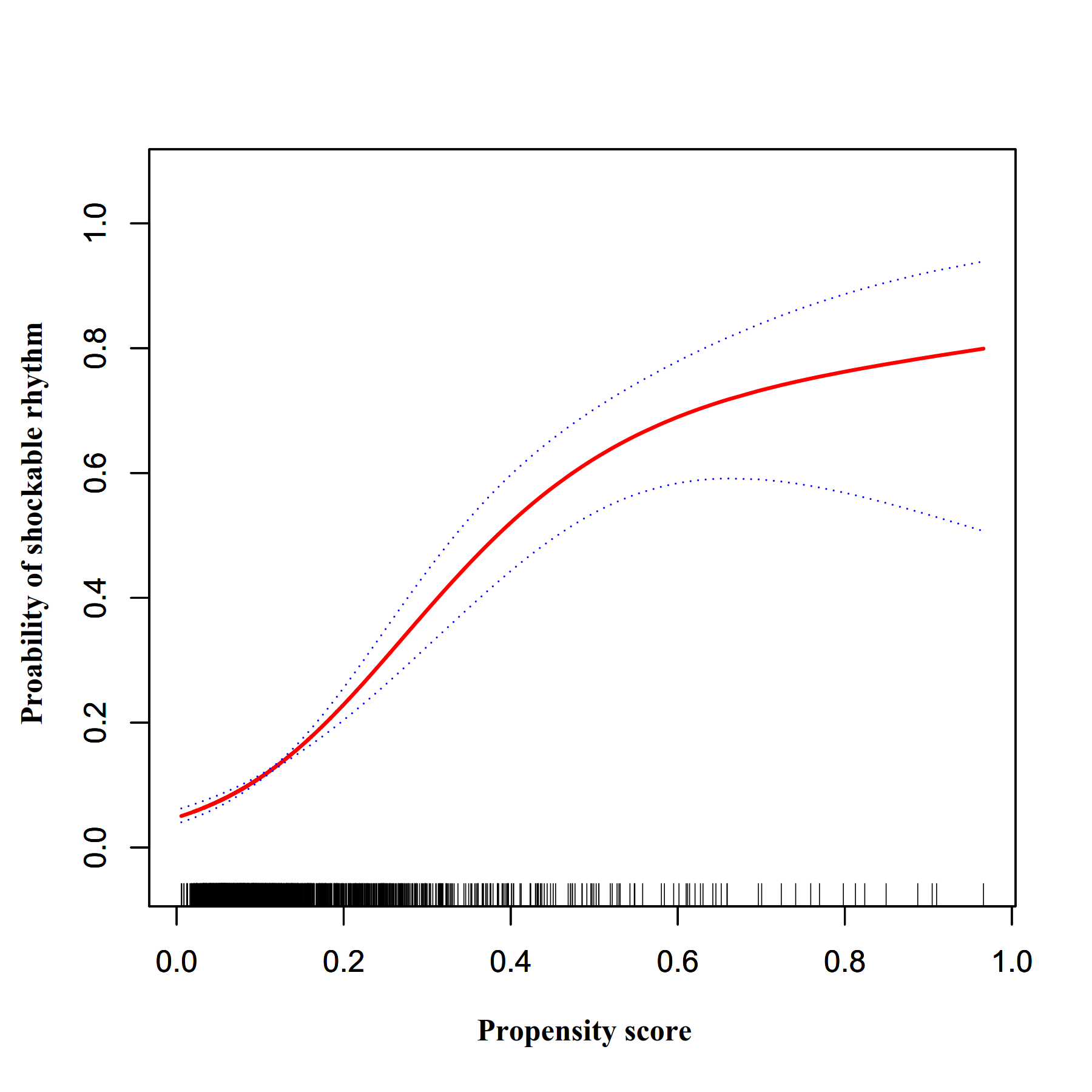


**Figure S1 The relationship between propensity score and initial cardiac rhythm in participants with IHCA.**

Figure S1 showed that a higher PS was linked to a higher probability of shockable rhythm in individuals with IHCA.
